# Spinal decompression and radionuclide therapy for an unresectable FGF23 transmitted tumor causing cervical myelopathy: a case report and literature review

**DOI:** 10.3389/fonc.2026.1754565

**Published:** 2026-02-04

**Authors:** Shin Yokoyama, Hirokazu Shimizu, Akiko Yuno, Junki Takenaka, Naoto Wakabayashi, Shiro Watanabe, Ken Kuwahara, Masatake Matsuoka, Tomohiro Onodera, Norimasa Iwasaki, Akira Iwata

**Affiliations:** 1 Department of Orthopedic Surgery, Faculty of Medicine and Graduate School of Medicine, Hokkaido University, Sapporo, Japan; 2 Department of Musculoskeletal Oncology, NHO Hokkaido Cancer Center, Sapporo, Japan; 3 Department of Endocrinology and Metabolism, Kin-ikyo Chuo Hospital, Sapporo, Japan; 4 Department of Diagnostic Imaging, Graduate School of Medicine, Hokkaido University, Sapporo, Japan; 5 Department of Nuclear Medicine and Comprehensive Heart Failure Center, University Hospital Würzburg, Würzburg, Germany; 6 Department of Nuclear Medicine, Hokkaido University Hospital, Sapporo, Japan; 7 Department of Pathology, NHO Hokkaido Cancer Center, Sapporo, Japan

**Keywords:** cervical myelopathy, FGF23 transmitted tumor, peptide receptor radionuclide therapy, spinal decompression, unresectable case

## Abstract

**Background:**

Tumor-induced osteomalacia (TIO) is a rare disorder characterized by hypophosphatemic osteomalacia, mainly caused by benign tumors with excessive secretion of fibroblast growth factor 23 (FGF23) and somatostatin receptor expression. Although complete excision is recommended, reports on treatment strategies for anatomically challenging surgical cases are lacking. We report an unresectable case of FGF23 transmitted tumor in the cervical vertebrae causing myelopathy, which was treated with surgical decompression combined with radionuclide therapy.

**Case presentation:**

A 52-year-old woman presented to another hospital with complaints of knee pain. After confirming abnormal tracer uptake at the C7 vertebrae using somatostatin receptor scintigraphy and an elevated serum FGF23 level (>800 pg/mL), TIO was diagnosed 7 years after the initial presentation. Gait disturbance occurred 10 years after the initial presentation; therefore, the patient was referred to our department. Magnetic resonance imaging revealed a tumor with spinal cord compression and vertebral artery encasement, making complete resection impossible. Gait disturbance improved after spinal decompression with partial resection of the tumor. Peptide receptor radionuclide therapy targeting somatostatin receptors was initiated 2 years after surgery. Serum phosphate levels normalized, and the tumor size remained stable after the initiation of PRRT. Ambulation was maintained without joint pain recurrence at 3 years after surgery.

**Conclusions:**

The current literature on FGF23 transmitted tumors in the cervical spine includes six cases treated with definitive local therapy. This case suggests an alternative option for unresectable FGF23 transmitted tumor in the vertebrae, causing spinal myelopathy.

## 1 Introduction

Tumor-induced osteomalacia (TIO) is a rare paraneoplastic syndrome attributable to the overproduction of fibroblast growth factor 23 (FGF23) by bone or soft tissue tumors, with an estimated prevalence of 0.43 per 100,000 adults (1). Benign mesenchymal tumors in various regions of the body produce FGF23 and phosphatonins in patients with TIO, leading to hypophosphatemia via reduced renal reabsorption of phosphate and defective bone mineralization, resulting in deformities and pain (1, 2).

Because of the nonspecific nature of the symptoms and small size of the tumor, diagnosing such cases without delay and localizing the tumor are often difficult (3). When detection using conventional modalities fails, somatostatin receptor positron emission tomography/computed tomography, which is a more sensitive tool (pooled detection rate of 87.6%), can be used to identify the tumor causing TIO (4–7).

The first-line treatment for TIO is curative local definitive therapy (8), and alternative therapies include phosphate, active vitamin D, and monoclonal antibodies against FGF23 (1). Recent case reports have suggested peptide receptor radionuclide therapy (PRRT) targeting somatostatin receptors as a potentially feasible treatment for FGF23 transmitted tumor (9–11).

Although a few cases of FGF23 transmitted tumor in the cervical spine are available in PubMed, all of them were treated with local definitive therapy (12–16). However, surgical resection of a tumor in the cervical vertebrae is challenging, especially when it compresses the spinal cord and/or vertebral artery. Herein, we report a case of an unresectable FGF23 transmitted tumor causing cervical myelopathy that was treated with spinal decompression and partial resection, followed by PRRT. The patient was ambulatory without joint pain because the findings showed that the tumor size remained stable and serum phosphate levels normalized after the initiation of PRRT. To the best of our knowledge, this is the first report to describe an effective treatment for unresectable FGF23 tumors with cervical cord compression.

## 2 Case description

A 52-year-old woman presented to a local orthopedic clinic with bilateral knee pain. The pain extended to both the ankles. Blood tests at the age of 54 years revealed hypophosphatemia with a low ratio of the tubular maximum reabsorption of phosphate (TmP) to the glomerular filtration rate (GFR). The patient was subsequently referred to the endocrinology department, where she was diagnosed with hypophosphatemic osteomalacia. Oral phosphate supplementation and active vitamin D analogs were initiated, resulting in partial improvement of knee pain.

**Figure 1:**
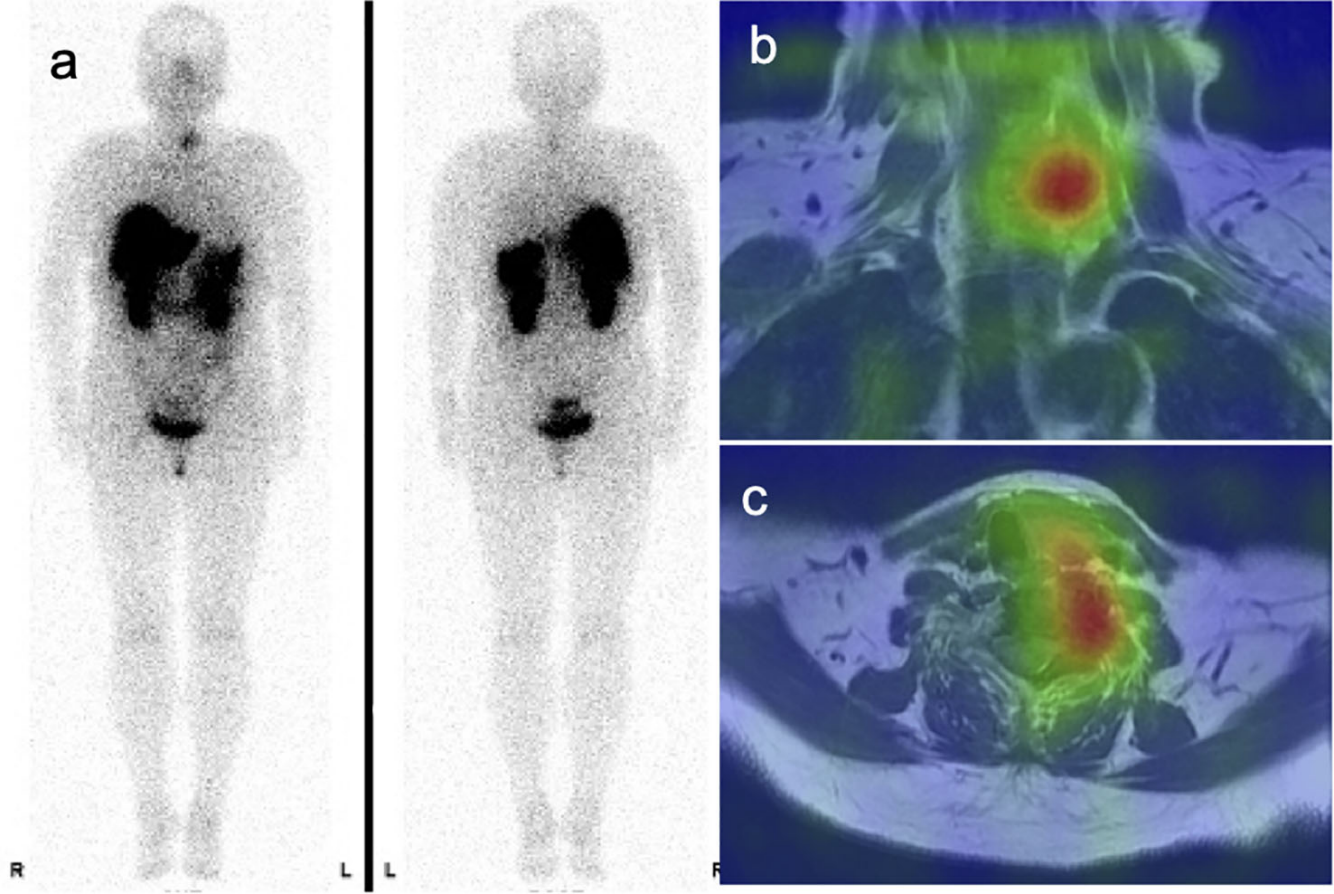
Diagnostic imaging findings. Somatostatin receptor scintigraphy with high sensitivity for diagnosing tumor-induced osteomalacia and detecting a positive lesion found radiotracer uptake in the ventral aspect of the C7–T1 vertebrae **(a–c)**.

Mild symptoms persisted 7 years after the initial presentation. An elevated serum FGF23 level was detected (more than 800 pg/mL, normal range of intact FGF23 by chemiluminescent enzyme immunoassay: 19.9 pg/mL–52.9 pg/mL), although the specific value could not be determined at this time. Because an FGF23 transmitted tumor was suspected, somatostatin receptor scintigraphy and magnetic resonance imaging (MRI) were performed, revealing a solitary lesion with abnormal tracer uptake at the C7 vertebrae (**Figure 1**). Therefore, TIO was suspected based on the diagnostic criteria (17, 18). Although complete surgical resection is the standard treatment, it poses a high risk of severe nerve injury and functional impairment, rendering it unfeasible. Consequently, conservative therapy, including oral phosphate supplementation and active vitamin D analogs, was continued.

Eight years after the diagnosis, anti-FGF23 monoclonal antibody therapy was initiated. However, no improvement in persistent symptoms was observed, and bone scintigraphy revealed increased uptake in multiple areas. Consequently, the therapy was discontinued after 10 months of treatment.

**Figure 2:**
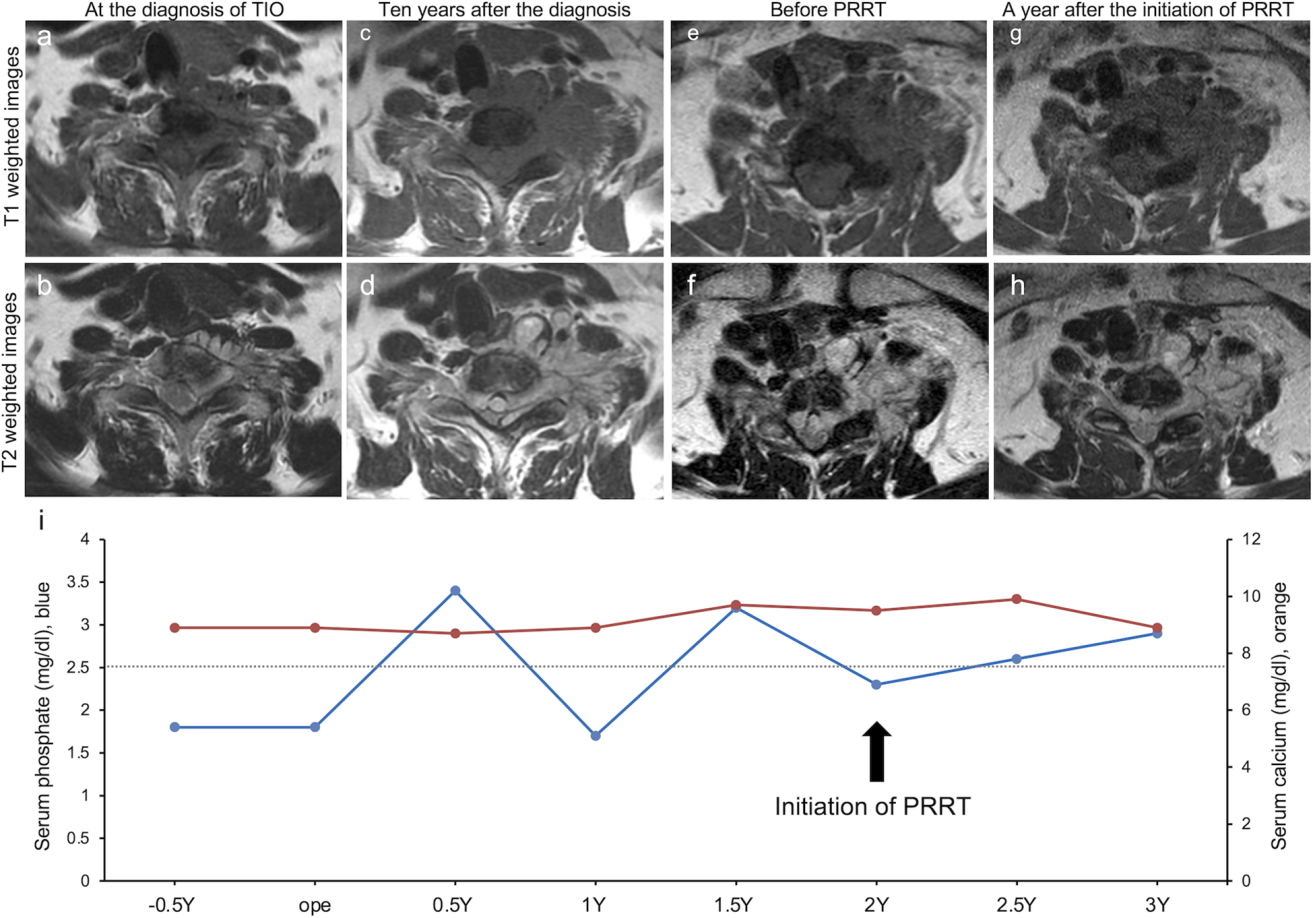
Longitudinal findings of medical images and laboratory tests. **(a, b)** Axial T1-weighted and T2-weighted imaging at the time of the diagnosis of tumor-induced osteomalacia (TIO) showing a 37-mm × 26-mm lesion at the right ventral aspect of the C7 vertebra. **(c, d)** Axial T1-weighted and T2-weighted imaging 10 years after the diagnosis showing a 56-mm × 39-mm lesion and marked tumor enlargement with extension into the spinal canal and compression of the spinal cord. **(e, f)** Cervical magnetic resonance imaging (MRI) (axial T1-weighted and T2-weighted images) immediately before peptide receptor radionuclide therapy (PRRT) initiation showing a 57-mm × 34-mm residual tumor. **(g, h)** Cervical MRI (axial T1-weighted and T2-weighted images) One year after PRRT initiation showing a 53-mm × 28-mm lesion. **(i)** Longitudinal laboratory tests of serum phosphate levels (blue, normal range: 2.5 mg/dL–4.5 mg/dL) and serum calcium levels (orange, normal range 8.5 mg/dL–10.2 mg/dL). Y, year(s).

Ten years after the initial diagnosis, the patient was referred to our department with progressive difficulty in ambulating. Among other symptoms, clumsiness dominated the bilateral ring and little fingers, and sensory disturbance involved all four extremities and the trunk. Neurological examinations showed an AISA impairment scale (AIS) Grade C. These findings were consistent with cervical myelopathy at the C8 level. Additional laboratory tests indicated elevated serum FGF23 (13,200 pg/mL), low serum phosphorus, normal serum calcium, elevated intact parathyroid hormone, normal 1,25 dihydroxy vitamin D, and elevated alkaline phosphatase levels. MRI revealed tumor enlargement with intradural extension at the C7-Th1 level, causing circumferential spinal cord compression and vertebral artery involvement (**Figures 2A–D**).

**Figure 3:**
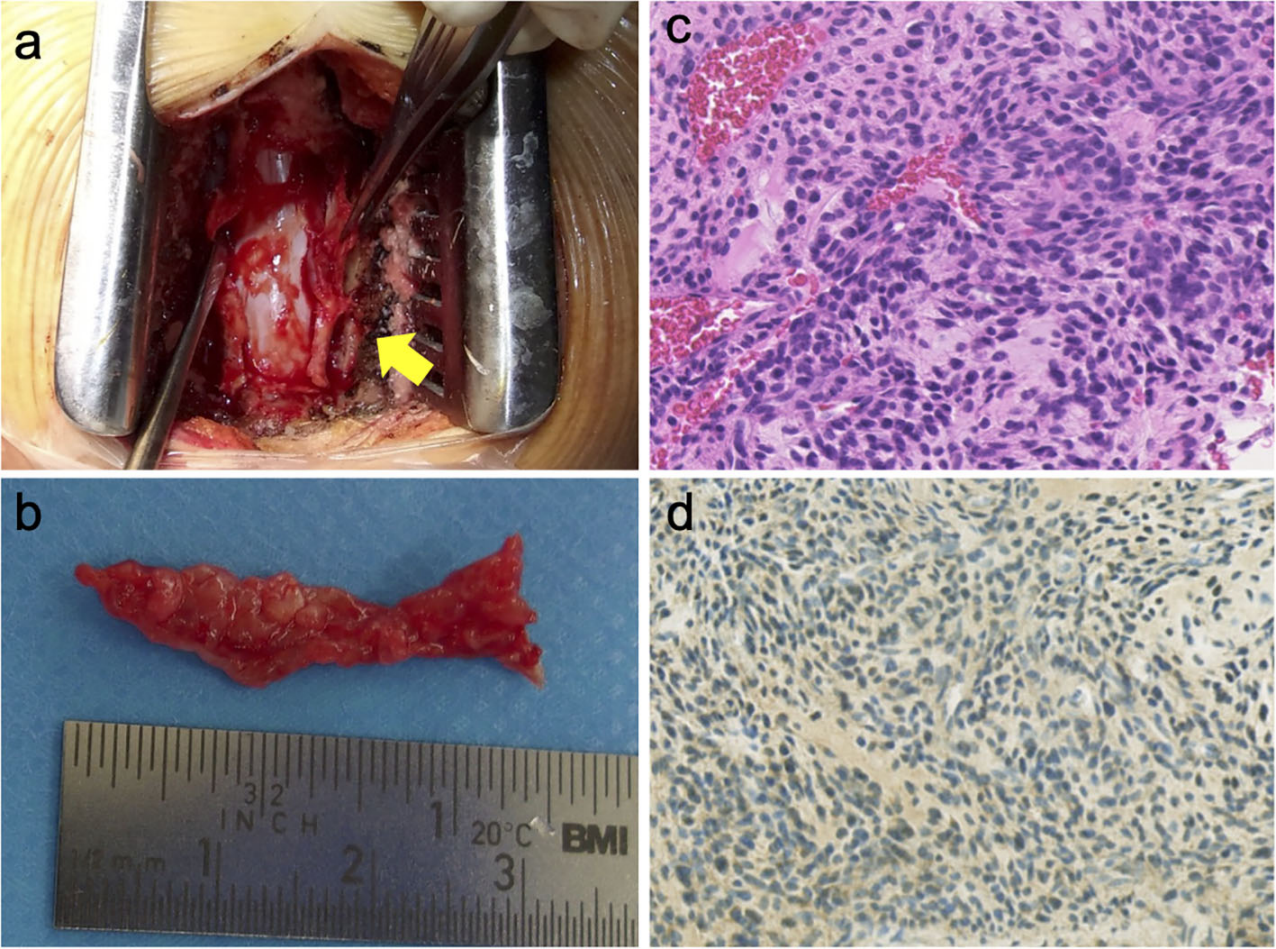
Intraoperative findings, gross specimen, and pathological images. **(a)** Intraoperative image showing partial resection of the intradural–extramedullary tumor compressing the spinal cord followed by laminoplasty. **(b)** Gross appearance of the resected tumor specimen. **(c)** Hematoxylin and eosin staining showing spindle-shaped tumor cells with densely proliferating relatively uniform round nuclei without mitotic figures or coagulative necrosis. **(d)** Immunohistochemical staining for CD56 demonstrating diffuse positive cytoplasmic expression.

Therefore, we planned decompression with partial resection. Laminectomy of C6–T1 was performed, and the intradural–extramedullary tumor was partially resected to decompress the spinal cord (**Figures 3A, B**). Histopathological examination revealed densely proliferating spindle-shaped tumor cells with relatively uniform round nuclei, without mitotic figures or signs of coagulative necrosis. Immunohistochemical analysis was negative for sarcoma-specific markers and positive for CD56 (**Figures 3C, D**). These histological findings were consistent with those of a phosphaturic mesenchymal tumor (PMT). Based on the diagnostic criteria for TIO (17, 18), this patient was diagnosed with TIO according to the clinical presentation of bone pain without a family history, laboratory findings including hypophosphatemia, low Tmp/GFR ratio, and markedly elevated FGF23 levels, solitary tumor localization confirmed by somatostatin receptor scintigraphy, and histological findings.

One month postoperatively, the patient’s status improved to AIS Grade D, and the patient was discharged with a walking aid. Recovery of gait stability and hand dexterity was noted, supporting a causal relationship between mechanical spinal cord compression by the tumor and the observed myelopathy. Additional local therapy was considered, but external beam radiotherapy was not indicated because the proximity of the tumor to the spinal cord was a limiting factor for treatment.

One year post-surgery, laboratory tests showed increased serum FGF23 levels (23,300 pg/mL). Because the tumor was positive for somatostatin receptor expression, PRRT with ^177^Lu-dotatate was initiated 2 years postoperatively. Three treatment cycles were administered over a period of several months. MRI revealed stable lesions in the residual tumor (**Figures 2E–H**). Longitudinal laboratory findings showed that the serum phosphate levels normalized at the follow-up examination 3 years postoperatively (**Figure 2I**). Serum FGF23 levels were not measured after PRRT initiation due to health insurance-related limitations. The patient remained ambulatory without bone or joint pain recurrence and exhibited stable neurological functions.

## 3 Discussion and review of the literature

We present a case of an unresectable FGF23 transmitted tumor causing cervical myelopathy that was effectively treated with spinal decompression and partial resection, followed by PRRT. The patient was ambulatory without joint pain 3 years post-surgery; furthermore, the tumor size remained stable, and serum phosphate levels normalized.

This case comprised a tumor at the cervical spine that caused difficulty in walking, and neurological examinations showed cervical myelopathy at the C8 level and AIS Grade C. Because the lesion compressed the vertebral artery and cervical cord, definitive local therapy was infeasible; therefore, decompressive partial resection was performed. Consequently, the patient was discharged with a walking aid, and her status improved to AIS Grade D at 1 month postoperatively. Based on the diagnostic criteria for TIO (17, 18), the patient was diagnosed with TIO according to the clinical presentation of bone pain without a family history; laboratory findings including hypophosphatemia, low Tmp/GFR ratio, and elevated FGF23 levels; solitary tumor localization confirmed by somatostatin receptor scintigraphy; and histological findings compatible with PMT.

While biomedical parameters are often rapidly corrected upon surgical removal of the TIO-causing tumor (1), surgical resection may not always be indicated, as in our case. The findings of our case demonstrated the normalization of serum phosphate levels and stable lesions of the residual tumor after PRRT initiation, suggesting therapeutic efficacy from biochemical and radiological perspectives. Serum FGF23 levels after the initiation of PRRT could not be determined owing to health insurance-related limitations in our country.

We searched MEDLINE for English publications using the following keywords: “FGF23,” “cervical spine,” and “phosphaturic mesenchymal tumor.” Only five cervical FGF23-producing PMTs have been reported in patients aged 52–71 years (**Table 1**) (12–16). The duration between onset and diagnosis ranged from 1 year to several years. Four patients presented with myelopathy. All patients exhibited hypophosphatemia and high FGF23 levels. Positron emission tomography/computed tomography and MRI enabled the localization of the tumor. Histological examination confirmed PMT and PMT with mixed connective tissue. Four patients underwent total surgical resection, and one patient underwent radiation as local definitive therapy.

**Table 1:** Reported cases of cervical spine FGF23 transmitted tumor.

Authors	Sex/Age at surgery	Level	Symptoms	Duration before diagnosis	Biochemistry	Diagnostic modality	HPE	Treatment	Follow-up and outcome
Akhter et al. (13)	52 M	C5	Bone pain, fractures	1 year	Low phosphate,	PET	PMT-MCT	C5 corpectomy and anterior and posterior fixation	1 year NSA
					low vitamin D,				
					elevated ALP				
Nakamura et al. (14)	72 M	C5	Weakness, spasticity, fractures	2 years	Low phosphate, elevated ALP	MRI	PMT	GTE and C4–C6 corpectomy and anterior spinal fusion	5 years NSA
Agarwal et al. (15)	52 M	C2	Bone pain, fractures, weakness	4 years	Low phosphate,	PET/CT	PMT-MCT	Hemilaminectomy and GTE	1.5 years NSA
					low vitamin D,				
					elevated ALP				
Agarwal et al. (15)	71 M	C2	Weakness, bone pain	Several years	Low phosphate, elevated FGF23	PET/CT MRI	PMT	Laminectomy and GTE	2 years NSA
Hockemeyer et al. (16)	65 M	C2	Bone pain, weakness,	Several years	Low phosphate, elevated FGF23	PET/CT, MRI	PMT	SBRT,	0.5 years NSA
								subsequent cement augmentation	
Yokoyama et al. (present case)	69 F	C7	Bone pain	7 years	Low phosphate, elevated FGF23	SRS MRI	PMT	PR and PRRT	3 years

CT, computed tomography; F, female; GTE, gross total excision; HPE, histopathological evaluation; M, male; MCT, mixed connective tissue; MRI, magnetic resonance imaging; NA, missing data/data not available for review; NSA, normalization of serum abnormalities; NR, not reported; PET, positron emission tomography; PMT, phosphaturic mesenchymal tumor; PR, partial excision; PRRT, peptide receptor radionuclide therapy; SBRT, stereotactic body radiation therapy; SRS, somatostatin receptor scintigraphy.

Medical management has become the primary treatment for inoperable cases. Conventional therapies include oral phosphate salts and active vitamin D analogs to correct hypophosphatemia (19, 20). Additionally, a monoclonal antibody that directly inhibits FGF23 has been introduced and approved for TIO (20). This antibody blocks the interaction between FGF23 and the fibroblast growth factor receptor 1–Klotho receptor complex, thus restoring phosphate reabsorption and 1,25(OH)_2_D_3_ synthesis in the renal proximal tubules (21). Thus, our patient was resistant to conservative therapy.

Most PMTs express somatostatin receptor 2 (22, 23), making PRRT feasible. ^177^Lu-dotatate binds to somatostatin receptor 2-positive cells, delivering β radiation to induce apoptosis (24). Three case reports have reported clinical or biochemical improvement in unresectable PMTs (9–11). Although limited, these results support PRRT as a promising treatment option for refractory or recurrent diseases. In our case, decompression relieved myelopathy, and PRRT produced radiologically stable status. Cervical PMTs are surgically demanding because of their proximity to vital neurovascular areas; therefore, combining limited resection with decompression radionuclide therapy appears to be a safe and effective strategy. Long-term data are scarce; therefore, continued imaging and biochemical monitoring are essential for these patients.

## 4 Conclusion

We encountered a rare case of unresectable cervical PMT with myelopathy that was successfully managed using partial decompression and postoperative ^177^Lu-dotatate therapy. During follow-up, the residual tumor was smaller, the patient was ambulatory, and no biochemical recurrence was observed. This case highlights that treatment comprising both surgical and molecular-targeted therapies can achieve durable control of anatomically unresectable cervical PMTs and is a viable alternative when radical resection and radiotherapy are contraindicated.

## Data Availability

The raw data supporting the conclusions of this article will be made available by the authors, without undue reservation.
